# Accessory liver lobe in the right thoracic cavity

**DOI:** 10.1002/jgf2.546

**Published:** 2022-04-06

**Authors:** Kousuke Ihara, Hiroki Isono

**Affiliations:** ^1^ Department of General Medicine HITO Medical Center Ehime Japan

**Keywords:** accessory liver lobe, hepatocellular carcinoma

## Abstract

A healthy 42‐year‐old man presented to the hospital because of chest radiography performed during a medical checkup revealed a tumor. Contrast‐enhanced computed tomography showed a tumor of 5 cm diameter just above the right diaphragm with blood flow from the portal vein. The patient was diagnosed with accessory liver lobe (ALL). No finding suggested malignancy, and he is being followed up. ALLs are usually found in the abdominal cavity, but they can also be found in the thoracic cavity. Although ALL is rare, it should be considered in patients presenting with intrathoracic tumors.

## Graphical abstract

A healthy 42‐year‐old man presented to the hospital because chest X‐ray taken during a medical checkup had revealed a tumor. He was diagnosed with accessory liver lobe (ALL). No findings suggested malignancy, and he is being followed up.Although ALL is rare, it should be kept in mind in patients presenting with intrathoracic tumor.

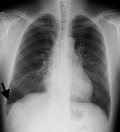

## 1 INTRODUCTION

Accessory liver lobe (ALL) is a congenital ectopic liver tissue, which is mainly due to embryonic dysplasia. ALL was first described in 1767.^1^ There are two types of ALL: an accessory lobe that is joined to normal liver tissue and an accessory lobe that is completely separated. Because completely separated ALL is rarely seen clinically and is difficult to diagnose non‐surgically, it is easily missed or misdiagnosed.^2^ Here, we report a case of ALL in the right thoracic cavity.

## 2 CASE PRESENTATION

**FIGURE 1:**
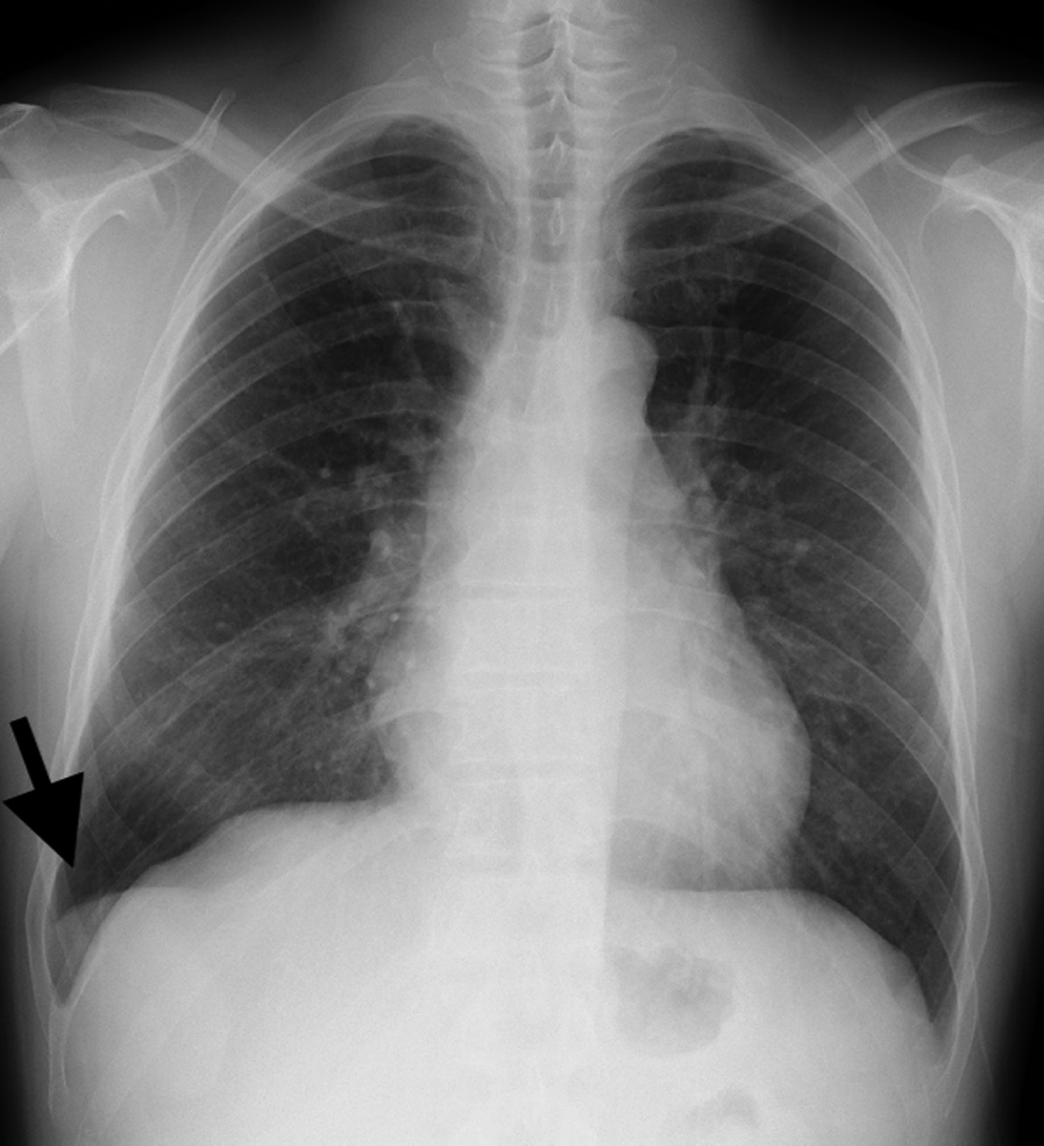
Chest radiograph shows a mass in the lower right thorax (arrow). The border of the right diaphragm is clearly seen

**FIGURE 2:**
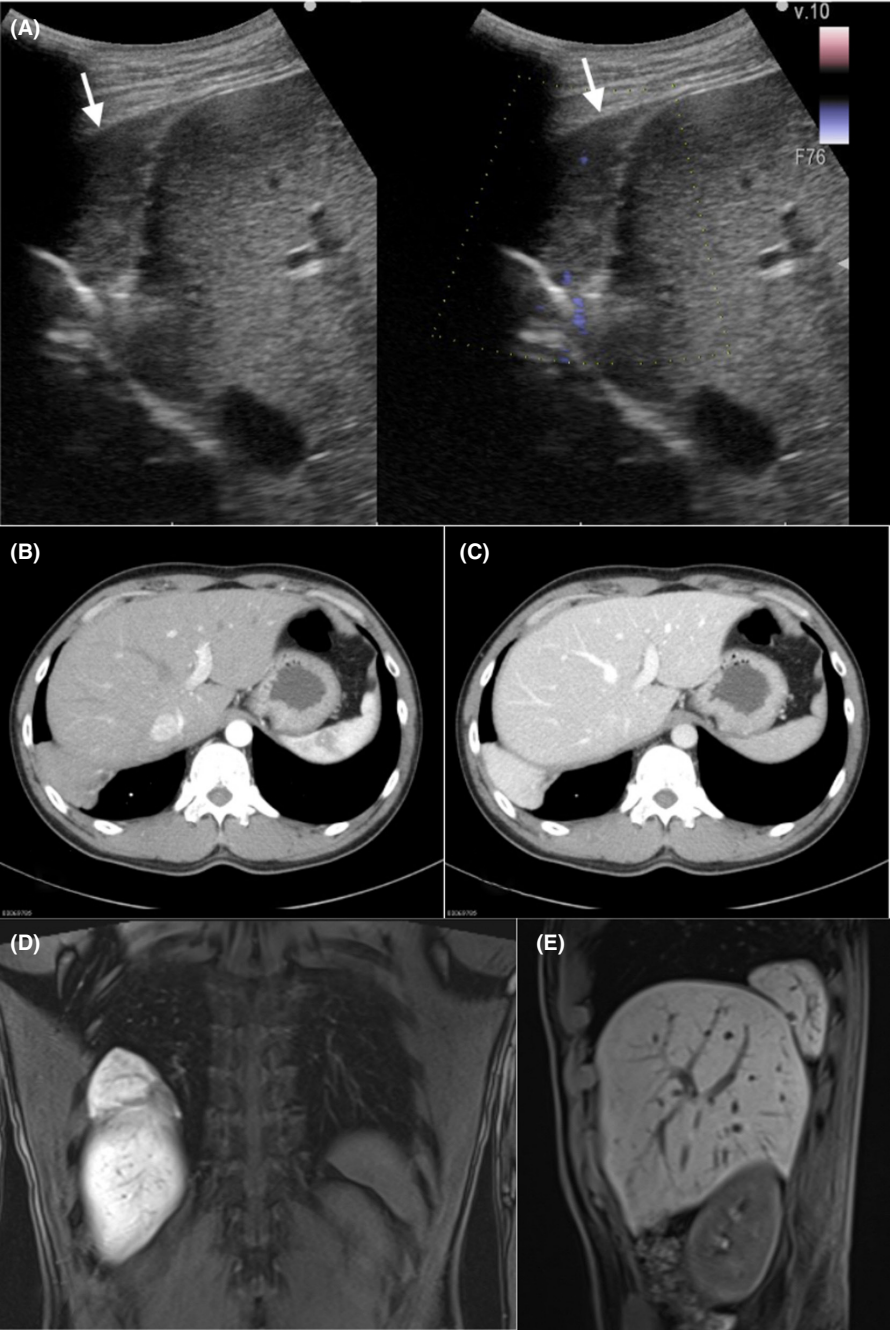
Abdominal ultrasonography (A). The mass is seen in the right costal region (arrow). The mass seems to be separated from the liver, but the echogenic level is similar to that of the liver. Vascular structures are seen inside the mass. Contrast‐enhanced computed tomography. (B): arterial phase. (C): late portal phase. The mass is seen in the lower right thorax. Blood from the mass flows into the portal vein, but no afferent arteries are visible. Its pattern of contrast uptake is similar to that of the liver. Contrast‐enhanced magnetic resonance imaging in the hepatocellular phase. (D): coronal view. (E): sagittal view. The mass is seen in the lower right thorax. Its pattern of contrast enhancement is similar to that of the liver

A healthy 42‐year‐old man presented to the hospital because his chest radiograph obtained during a medical checkup revealed a mass overlapping the right diaphragm (Figure 1). He was asymptomatic, and his laboratory test results revealed no abnormality. Abdominal ultrasonography showed a solid mass in the right costal region (Figure 2A). Contrast‐enhanced computed tomography showed a mass measuring 5 cm in diameter just above the right diaphragm. Blood from the mass flowed into the portal vein, but no afferent arteries were visible. The pattern of contrast uptake of the mass was similar to that of the liver (Figure 2B,C). Contrast‐enhanced magnetic resonance imaging showed a contrast‐enhancing effect in the hepatocellular phase (Figure 2D, E). A structure previously considered to be a lung tumor appeared to be part of the liver within the thoracic cavity. Based on these imaging findings, the patient was diagnosed with an ALL. Because no finding suggested malignancy, the patient did not undergo surgery and is being followed up.

## 3 DISCUSSION

The first lesson of this case is the importance of proper detection of tumors in contact with the diaphragm on a chest radiograph. This is because superimposed normal structures covered by the diaphragm are the major contributing factor to overlooked lesions.^3^ Second, intrathoracic ALL can be diagnosed by radiologic investigations. Most ALLs are located in the abdominal cavity, but a few may be located in the thoracic cavity. In a report of 76 ALL cases by Akura et al., all ALLs were located in the abdominal cavity, with the most common location (65%) being around the gallbladder.^4^ Morita et al. described 39 cases of hepatocellular carcinoma arising from ALL and mostly occurring in the abdominal cavity; only one case involved the thoracic cavity.^5^ Patients with ALL who are asymptomatic or who do not have serious complications do not require treatment. Complications include torsion, infarction, traumatic rupture, or hepatocellular carcinoma.^6^, ^7^ In cases of thoracic ALL, thoracotomy is occasionally performed because the lesion can be mistaken for a lung tumor, and ALL is diagnosed only postoperatively.^8^ As illustrated by this case, appropriate radiologic investigations may obviate the requirement of surgery. ALL should be considered in the differential diagnosis of patients presenting with intrathoracic tumors.

## CONFLICT OF INTEREST

The authors have stated explicitly that there are no conflicts of interest in connection with this article.

## AUTHOR CONTRIBUTIONS

All authors meet the ICMJE authorship criteria.

## PATIENT CONSENT

The patient has provided free written informed consent for the publication of this manuscript.

## INFORMED CONSENT

The patient provided written informed consent.
